# Challenges and Initiatives in Diversity, Equity and Inclusion in Cancer Molecular Imaging

**DOI:** 10.3389/fonc.2021.638692

**Published:** 2021-04-09

**Authors:** Heike E. Daldrup-Link, Giuseppe Esposito, Zaver M. Bhujwalla

**Affiliations:** ^1^ Department of Radiology, Stanford University School of Medicine, Stanford, CA, United States; ^2^ Department of Radiology, Georgetown University Hospital, Washington, DC, United States; ^3^ Department of Radiology, Johns Hopkins University School of Medicine, Baltimore, MD, United States

**Keywords:** diversity & inclusion, cancer molecular imaging, equity, radiology, STEM - science technology engineering mathematics

## Abstract

A diverse biomedical workforce is essential to achieve excellence in patient care, clinical translational, and basic research. Diversity, equity, and inclusion challenges in cancer molecular represent a combination of the challenges facing the science, technology, engineering, and mathematics (STEM) field, and challenges in Radiology and Nuclear Medicine. Although there is a growing awareness of conscious and unconscious bias that negatively affect the cancer imaging world, many challenges remain such as overcoming barriers to entry into the pipeline, avoiding program dropout, and providing long-term career prospect. The COVID-19 pandemic has resulted in a significant setback and further highlighted problems faced by women and underrepresented minorities. In this perspective, we have identified some of the challenges faced and highlighted ongoing and future initiatives to address these challenges.

## Introduction

Diversity of our population in terms of geographic and economic backgrounds, skin color, age, gender, sex and sexuality is a major hallmark of our century. Currently, racial and ethnic minorities make up nearly 40% of the United States population. By 2050, non-white people will represent more than 50% of the general population (www.census.gov). As a consequence, building a diverse biomedical workforce is essential to excellence in patient care, clinical translational, and basic research (1, 2). Diverse teams are better at solving complex problems and relate better to the general public. Diversity has been linked to improved access and quality of care for minorities and female patients (3, 4). Female and Underrepresented in Medicine (URiM) faculty serve as important resources for patients from diverse backgrounds and important role models and mentors to minority trainees (5).

The cancer molecular imaging field represents a fusion of expertise in various disciplines such as molecular biology, engineering, chemistry and computational sciences. Because molecular imaging is increasingly being integrated into diagnostic imaging and imaging directed treatments and interventions, clinical radiologists and nuclear medicine physicians form an integral part of the field. The challenges for diversity, equity and inclusion in this field therefore represent a combination of the challenges facing the science, technology, engineering and mathematics (STEM) field, and challenges in Radiology and Nuclear Medicine.

The past decade has clearly witnessed a growing awareness of conscious and unconscious bias that negatively affect the entire Radiology workforce. Among training programs of 20 sub-disciplines in clinical medicine, diagnostic radiology ranks 17th in female and 20th in underrepresented minorities (URM) representation (https://doi.org/10.1148/radiol.13130101). Thus, there is an urgent need for Radiology to address its dire underrepresentation of female and racial/ethnic representation in both preclinical molecular imaging research and clinical-translational molecular imaging. Overcoming barriers to entry into the pipeline, avoiding program dropout and providing long-term career prospects are all important challenges towards the goal of successful careers of those who are underrepresented in the field. The COVID-19 pandemic has presented additional barriers and fractures in our work-force, and further highlighted problems faced by women and URM. While the switch to virtual academic interactions, remote teaching and training, and virtual radiology, has allowed more flexibility, overall the disruption to childcare and school routines has significantly negatively impacted women and URM. As discussed in a recent study (6), URM researchers across the board from students and trainees to faculty have faced multiple challenges due to the pandemic due to wide-ranging issues such as a disparate loss of opportunities for students and trainees, and the smaller research programs of URM researchers making them vulnerable with the shutdown of research operations.

## Challenges in Diversity, Equity and Inclusion

The importance of diversity in STEM to increase talent and problem solving, and to improve long-term economic growth and global competitiveness, is clearly recognized (7). While significant inroads have been made in actively supporting diversity and inclusion in the STEM field, published numbers indicate that a disproportionately low number of women and black, Latinx and Native Americans enter the STEM fields, and specifically, Radiology. Some of the barriers include socio-economic disparities that can lead to inability to pay for colleges, reduced access to advancement placement courses, and challenges arising from associating with different demographics and cultural majorities (A Guide for Minorities in STEM: Increasing Workplace Diversity, 2020). In academic and research centers, while discrepancies in the numbers of women and URM graduate students, fellows and junior faculty is decreasing, women and URM numbers significantly dwindle in the transition to senior faculty and leadership positions. The importance of role models and mentors that demonstrate diversity in leadership roles can contribute significantly to reducing disenfranchisement of women and URM in STEM.

Diversity of trainees and faculty in the field of Clinical Cancer Molecular Imaging is important because cross-cultural communication and access to a diverse group of physicians leads to better health outcomes (8). Cultural competence is the ability to understand and effectively communicate with people from different cultures (9). Healthcare providers with broad language competence (10–13) and an understanding of culture-specific concepts (14–16), positively impact medical care by creating closed-loop communications, reducing medical errors and enhancing positive health outcomes.

However, despite significant efforts of academic institutions, the representation of female and racial/ethnic minority faculty members in Academic Radiology departments remains low, especially in higher ranks and leadership roles. Cater et al. reported that female radiologists comprise 33.5% of all radiologists worldwide, with the lowest proportion in the United States (27.2%) (17). The problem starts with our pipeline: West et al. reported that all radiology fellowship programs in the U.S. suffer from variable levels of gender and ethnic disparities (3). Reported relative numbers of female faculty were 15.4% in interventional radiology (18), 23% in neuroradiology (19), and 30.66% in musculoskeletal radiology (20). Of particular concern is the decreasing relative number of females compared to males within higher academic ranks and in radiology leadership positions (19, 20). For example, a study by Ahmadi et al. reported that 87.5% of neuroradiology leadership positions were occupied by men compared to 12.5% occupied by women (19). Even in breast imaging, where female faculty predominate, no correlation was noted between female gender and leadership positions (p = 0.57) (20). Black and non-white Hispanic faculty represent less than 10% of Radiology faculty nationwide, with reported proportions in the order of 2% Black and 6.2% Hispanic faculty (18).

Underrepresented minorities in Radiology, especially black and hispanic people, are used to being the “only” in their cohort, program, or department. Black and hispanic people remain starkly underrepresented in almost all STEM graduate programs, and this is only exacerbated as we consider postdoctoral fellows, faculty, and leadership roles. In addition to the general challenges that face all trainees in the fast-paced and often competitive environment of academia, this lack of representation and visibility can often leave URM students and other marginalized groups feeling like this space is not meant for them. Unilateral hierarchies represent the root cause for many acts of microaggression and disparities reported by racial/ethnic URM in STEM fields. Therefore, increasing the quota/proportion of underrepresented minorities alone is not enough. We need to increase the representation of qualified underrepresented minorities in leadership roles in order to ensure that every team member has an advocate at the leadership table, when decisions are being made. Introducing powerful advocates for everyone will reduce the risk of discrimination and harassment at the workplace and provide our students and junior faculty with a diverse set of role models.

## Initiatives in Cancer Molecular Imaging

The infrastructure to meet these challenges should combine top-down and grass roots approaches to increasing diversity and inclusion in the STEM and cancer molecular imaging fields. Programs that identify talented candidates early on, and continue to support them throughout their careers, would increase the success of women and URM, and build a cadre of leaders that would serve as role models for future generations. Financial support for such programs through philanthropy or other funding mechanisms is critically important.

Department leaders, scientific societies, and programs are recognizing the importance considering and including qualified women and underrepresented minorities in leadership training programs, committees, and consideration for awards and honors. The American College of Radiology (ACR) has established a Commission for Women and Diversity with the intent to celebrate diversity and actively promote inclusion at all levels of training, practice and leadership. The Society of Nuclear Medicine and Molecular Imaging (SNMMI) has established a Diversity, Equity and Inclusion Task Force with the goal to develop strategies to make all people feel that they belong to and can bring their true authentic self to their work place. These efforts are important, but should be expanded. Opportunities should be aggressively advertised to women and underrepresented minorities through appropriate venues, as in some instances, there may be an absence of awareness of these opportunities. There should be expanded training to raise awareness of conscious and unconscious bias. Biases, when these are identified, should be directly confronted and addressed (21). At the same time, evaluation biases need to be minimized by maximizing blinded reviews of research articles, grants and job applications. Dedicated courses and workshops focused on the importance of diversity and inclusion, and on identifying and eliminating conscious and unconscious bias are important to break down barriers to diversity and increase inclusion. The initiatives can be tailored to meet the needs of the situation. For instance, the stressors from the COVD-19 pandemic, in addition to the ongoing disparities regarding women and URM trainees, students, and faculty, have highlighted the urgent need for academic centers to implement corrective actions for all researchers and especially for women and URM. An excellent overview of strategies for pandemic-related researcher needs presented by Carr et al. (6) can also be adopted and implemented as general strategies to advance Diversity and Inclusion in academic centers. Creating internal bridge funding strategies, and developing guidelines for unbiased distribution of seed funds, may ameliorate, to some extent, the major set-back of the pipe-line and career advancement of URM and women that has occurred due to the pandemic.

### Trainees

Training in clinical molecular imaging provides a broad foundation for a wide range of career opportunities, ranging from Radiology faculty positions, research and management positions, radiation physicist positions and careers in computer sciences at Universities and the industry. Thus, our field is uniquely poised to impact diversity in STEM through pipeline programs. While graduate students and postdoctoral fellowship candidates can apply to a variety of training programs, none of these programs specifically address challenges and disparities experienced by trainees from URM backgrounds. Many R1 Universities receive more than 500 applications every year for less than 10 training spots provided by existing training programs. While the selection committees for these programs carefully review every application and consider trainees from all backgrounds, it is impossible to move the needle in terms of URM representation through these programs alone. The marked underrepresentation of Black and Hispanic students specifically would require influx of these students into the academic system at a higher rate. Adding training spots to existing training programs, perhaps through philanthropic initiatives, could impact the current underrepresentation of URM students in the field of molecular imaging more effectively by enabling an increased influx of URM trainees into our discipline. This increased number of URM trainees would lead to an increased availability of qualified candidates for faculty positions, and ultimately, increased representation of URM among Radiology faculty.

The imposter syndrome is experienced by high-achieving individuals who doubt their achievements. In a recent study conducted by our team at Stanford (22), we found that female and racial/ethnic minority status was strongly associated with self-reported imposter syndrome (p=0.006). By contrast, white male status was strongly associated with perceived recognition of their efforts (p=0.002). An imposter syndrome might prevent URM and female trainees to submit their scientific manuscripts to high impact journals, to apply for faculty positions at R1 Universities and to apply for leadership roles at different stages in their career. Dedicated mentoring, sponsorship and public acknowledgment of accomplishments can significantly improve the confidence of these individuals to try new responsibilities.

### Junior Faculty

Despite substantial monetary investments in gender and racial/ethnic equality programs, large proportions of minority faculty members drop out at the early and mid-career stage (23). Daily microaggressions can significantly impact the experience and long-term career success of underrepresented minority faculty. Examples include inappropriate comments or minority team members being relegated to mundane tasks. Intentional and unintentional prejudices can lead to biased formal evaluations of minority faculty. A highly effective approach to unbiased faculty evaluations would be to replace subjective evaluations by objective and measurable evaluation criteria, such as number of publications, impact factors and grants rather than “popularity scores”, which nurture and favor office politics over productivity. As mentioned earlier, evaluation biases should be directly addressed by implementing blinded reviews of research articles, and grant and job applications.

Many initiatives provide dedicated funding to support research efforts of junior faculty *via* reduced paylines or dedicated funding opportunities that are restricted to faculty at junior ranks. While specific support for junior faculty is certainly appreciated, many junior faculty find themselves stranded at the mid-career level, when these funding sources are not available to them anymore. This often leads to a dropout of highly qualified personnel after 6- or 7-figure investments by their institutions and/or tax payers. It is an unspoken reality that many academic faculty nationwide spend the vast majority of their time writing grants. This cannot be in the interest of medical innovation and discovery. As a community, we have to address this problem: We have to expose research-interested academic radiologists to a range of career options at an early career stage so that the field can leverage their skills rather than seed-funding blind ending careers. For clinical molecular imaging researchers with an interest in clinical-translational research, more long-term funding opportunities should be provided such that researchers can truly focus on research and discovery, without constant distractions by grant-writing duties and fears about their short or long-term job security. While this applies to all researchers, long-term career security is especially important for researchers from financially underserved communities. Members of financially affluent backgrounds may be more open to taking risks with regards to long-term financial insecurity. On the other hand, members from financially disadvantaged communities may put a higher emphasis on financial security, thereby excluding academic research jobs as viable career options.

### Established Investigators

Providing adequate resources and time for both clinical and research work is essential to maximize the value and excellence of clinical cancer molecular imaging. Our data at Stanford showed, in accordance with others, that female and racial/ethnic minority faculty reported less access to resources compared to male faculty (22). For example, several female faculty noted that significantly more intra-mural grants were assigned to male compared to female faculty. The vast majority of named professorships in Radiology and Molecular Imaging nationwide are assigned to men. This disparity has implications on productivity and leadership development. A study by McDonald et al. of faculty publication records at 4 large academic radiology centers found that male faculty had a significantly higher percentage of last author publications than female faculty (P <.0001), while female faculty had a significantly higher percentage of first author publications (P = .0025) (24). The first author is typically conducting the “hands on” experiments, while the last or senior author is often the division/group leader. It has been described that women are disproportionally doing the front-line work and “institutional housekeeping” while men disproportionally build their academic record as resource owners (25). It would be interesting to evaluate, if resource allocation precedes academic productivity or *vice versa*. In other words: Does promotion to a leadership role lead to increased productivity as a result of increased access to resources, which in turn is rewarded with further promotions. The composite of individual psychological experience of bias and lack of clearly defined promotion metrics, combined with lack of individualized support and guidance feeds this overall issue. Initiatives at a nationwide level should explore the efficacy of interventions that help to mitigate this problem. Likely individualized sponsorship would help greatly.

## Conclusion

An inclusive world with scientists and clinicians that represent all spectrums of our society is one of the most worthwhile and important goals of our century. The COVID-19 pandemic has clearly posed additional challenges in attaining this goal. These challenges have served to highlight the importance of global co-operation and inclusion, and our resilience as we work towards a better world. Academic and research institutions, medical and academic societies, medical practices have all had to adjust to the disruptive forces of the pandemic to bridge distances using virtual interactions for outreach, teaching and training. These virtual capabilities and tools can be exploited in molecular imaging to break down barriers to inclusion, and to access high level specialized education, so that opportunities can become equal for everyone, and for the field of clinical and research molecular imaging to benefit from a diverse, vibrant, global community.

## Data Availability Statement

The original contributions presented in the study are included in the article/supplementary material. Further inquiries can be directed to the corresponding author.

## Author Contributions

All authors have equally contributed to this perspective. All authors contributed to the article and approved the submitted version.

## Conflict of Interest

The authors declare that the research was conducted in the absence of any commercial or financial relationships that could be construed as a potential conflict of interest.
